# Predictors of functional outcome after thrombectomy for M2 occlusions: a large scale experience from clinical practice

**DOI:** 10.1038/s41598-023-45232-x

**Published:** 2023-10-31

**Authors:** Helge Kniep, Lukas Meyer, Gabriel Broocks, Matthias Bechstein, Helena Guerreiro, Laurens Winkelmeier, Caspar Brekenfeld, Fabian Flottmann, Milani Deb-Chatterji, Anna Alegiani, Uta Hanning, Götz Thomalla, Jens Fiehler, Susanne Gellißen, The German Stroke Registry – Endovascular Treatment (GSR – ET), Joachim Röther, Bernd Eckert, Michael Braun, Gerhard F. Hamann, Eberhard Siebert, Christian Nolte, Sarah Zweynert, Georg Bohner, Jörg Berrouschot, Albrecht Bormann, Christoffer Kraemer, Hannes Leischner, Jörg Hattingen, Martina Petersen, Florian Stögbauer, Boeckh-Behrens, Silke Wunderlich, Alexander Ludolph, Karl-Heinz Henn, Christian Gerloff, Jens Fiehler, Götz Thomalla, Anna Alegiani, Maximilian Schell, Arno Reich, Omid Nikoubashman, Franziska Dorn, Gabor Petzold, Jan Liman, Jan Hendrik Schäfer, Fee Keil, Klaus Gröschel, Timo Uphaus, Peter Schellinger, Jan Borggrefe, Steffen Tiedt, Lars Kellert, Christoph Trumm, Ulrike Ernemann, Sven Poli, Christian Riedel, Marielle Sophie Ernst

**Affiliations:** 1 Department of Diagnostic and Interventional Neuroradiology, University Medical Center Hamburg-Eppendorf, Martinistraße 52, 20246 Hamburg, Germany; 2 Department of Neurology, University Medical Center Hamburg-Eppendorf, Hamburg, Germany; 3 Department of Neurology, Asklepios Klinik Altona, Hamburg, Germany; 4 Asklepios Klinik Altona, Hamburg, Germany; 5 Bezirkskrankenhaus Günzburg, Günzburg, Germany; 6 Charité – Benjamin Franklin und Campus Charité, Berlin, Germany; 7 Charité - Campus Virchow Klinikum, Berlin, Germany; 8 Klinikum Altenburger Land, Altenburg, Germany; 9 Klinikum Lüneburg, Lüneburg, Germany; 10 KRH Klinikum Nordstadt, Hannover, Germany; 11 Klinikum Osnabrück, Osnabrück, Germany; 12 Klinikum R.d.Isar, Munich, Germany; 13 Sana Klinikum Offenbach, Offenbach, Germany; 14 UKE Hamburg-Eppendorf, Hamburg, Germany; 15 Uniklinik RWTH Aachen, Aachen, Germany; 16 Uniklinik Bonn, Bonn, Germany; 17 Klinikum Nürnberg, Nürnberg, Germany; 18 Uniklinik Frankfurt/ Main, Frankfurt, Germany; 19 Universitätsmedizin Mainz, Mainz, Germany; 20 Universitätsklinik Johannes Wesling Klinikum Minden, Minden, Germany; 21 Uniklinik München LMU, Munich, Germany; 22 Universitätsklinik Tübingen, Tübingen, Germany; 23 Universitätsmedizin Göttingen, Göttingen, Germany

**Keywords:** Stroke, Risk factors

## Abstract

Mechanical thrombectomy (MT) for acute ischemic stroke with medium vessel occlusions is still a matter of debate. We sought to identify factors associated with clinical outcome after MT for M2-occlusions based on data from the German Stroke Registry-Endovascular Treatment (GSR-ET). All patients prospectively enrolled in the GSR-ET from 05/2015 to 12/2021 were analyzed (NCT03356392). Inclusion criteria were primary M2-occlusions and availability of relevant clinical data. Factors associated with excellent/good outcome (modified Rankin scale mRS 0–1/0–2), poor outcome/death (mRS 5–6) and mRS-increase pre-stroke to day 90 were determined in multivariable logistic regression. 1348 patients were included. 1128(84%) had successful recanalization, 595(44%) achieved good outcome, 402 (30%) had poor outcome. Successful recanalization (odds ratio [OR] 4.27 [95% confidence interval 3.12–5.91], *p* < 0.001), higher Alberta stroke program early CT score (OR 1.25 [1.18–1.32], *p* < 0.001) and i.v. thrombolysis (OR 1.28 [1.07–1.54], *p* < 0.01) increased probability of good outcome, while age (OR 0.95 [0.94–0.95], *p* < 0.001), higher pre-stroke-mRS (OR 0.36 [0.31–0.40], *p* < 0.001), higher baseline NIHSS (OR 0.89 [0.88–0.91], *p* < 0.001), diabetes (OR 0.52 [0.42–0.64], *p* < 0.001), higher number of passes (OR 0.75 [0.70–0.80], *p* < 0.001) and intracranial hemorrhage (OR 0.26 [0.14–0.46], *p* < 0.001) decreased the probability of good outcome. Additional predictors of mRS-increase pre-stroke to 90d were dissections, perforations (OR 1.59 [1.11–2.29], *p* < 0.05) and clot migration, embolization (OR 1.67 [1.21–2.30], *p* < 0.01). Corresponding to large-vessel-occlusions, younger age, low pre-stroke-mRS, low severity of acute clinical disability, i.v. thrombolysis and successful recanalization were associated with good outcome while diabetes and higher number of passes decreased probability of good outcome after MT in M2 occlusions. Treatment related complications increased probability of mRS increase pre-stroke to 90d.

## Introduction

Over the past few years, several randomized trials have proven safety and efficacy of mechanical thrombectomy (MT) in large vessel occlusion (LVO) stroke, which are defined as occlusions in the intracranial internal carotid artery (ICA) and the M1 segment of the middle cerebral artery (MCA)^1^. For patients with LVO, MT has therefore been incorporated in the daily clinical practice of stroke centers, as well as in international guidelines^1–3^.

However, despite the substantial morbidity associated with medium vessel occlusions^4,5^ and emerging data suggesting that MT might also be safe and effective for medium vessel occlusions^4,6^ guidelines have not yet been clearly defined and MT has not been definitively established as standard of care. Due to the major advances in endovascular retriever and aspiration technology, MT has been recently emphasized by an international consensus as an encouraging option for medium and distal occlusions^7^ and is now increasingly performed in medium vessel occlusion strokes^8^. A recent meta-analysis of randomized trials (RCTs) on MT that also enrolled patients with M2 occlusions confirmed improved clinical outcome for patients receiving MT (n = 195) versus best medical management (n = 322)^9^. Furthermore, odds ratios for functional independence were reported for subgroups differentiated by perfusion mismatch and severity of initial symptoms. However, at present, the number of studies evaluating patient-specific factors associated with functional outcome of MT after medium and distal occlusions based on actual clinical experience is relatively low and included study populations are small^10,11^.

We therefore sought to identify baseline clinical, imaging, and MT factors associated with good clinical outcome after MT for M2 occlusions based on data from the German Stroke Registry-Endovascular Treatment (GSR-ET), a prospective multicenter registry representing real-world clinical experience.

## Methods

This multi-center registry study was approved in accordance with the Declaration of Helsinki^12^ by the leading ethics committee of this multi-center registry in Munich (chamber of physicians at Ludwig-Maximillians University LMU, Munich (689-15)). Approval by local ethics committees or institutional review boards was obtained for all participating sites according to local regulations. The analysis was conducted in accordance with the Transparent reporting of a multivariable prediction model for individual prognosis or diagnosis guidelines^13^. Patients, if capable, or their legal representatives, if available, were asked to provide written informed consent. All information was collected within clinical routine. Informed consent was obtained at the latest prior to the follow-up assessment 90 days after stroke.

### Patients

All patients with anterior circulation stroke prospectively enrolled in the German Stroke Registry-Endovascular Treatment (GSR-ET) between June 2015 and December 2021 were analyzed. GSR-ET is an ongoing, open label, prospective, multicenter registry of 25 sites in Germany collecting consecutive patients undergoing MT (NCT03356392). A detailed description of the GSR-ET study design and the major findings have been published recently^14,15^. The main inclusion criteria of GSR-ET are diagnosis of acute ischemic stroke due to large vessel occlusion, initiation of an endovascular procedure for treatment, and age ≥ 18 years, according to national guidelines. There are no exclusion criteria. For this analysis, all patients with primary M2 occlusion, availability of baseline clinical data, neurological status at admission and 90 days, and recanalization status were included. Patients with multiple occlusions were excluded. Occlusion location was determined according to GSR-ET protocol with differentiation of MCA occlusions into proximal and distal M1 occlusions and M2 occlusions based on admission imaging and DSA findings. The data that support the findings of this study are available from the GSR-ET but restrictions apply to the availability of these data, which are not publicly available. The corresponding author has access to the registry data acquired in the above-mentioned enrolment period.

### Statistics

Standard descriptive statistics were used for all study end points. Univariable distributions of metric variables were described with means/medians and interquartile range and categorical variables with absolute and relative frequencies. We aimed to identify baseline clinical, imaging, and MT factors associated with excellent functional outcome (defined as modified Rankin scale score (mRS) of 0–1 at day 90), good functional outcome (mRS of 0–2) and poor functional outcome/death (mRS of 5–6) at day 90. Successful recanalization was defined as Thrombolysis in Cerebral Infarction (TICI) ≥ 2b. Multivariable logistic regression analysis was performed to identify the independent predictors of excellent, good and poor functional outcome available at admission until directly after treatment. To further investigate the actual patient benefit associated with MT in M2 occlusions the increase of mRS pre-stroke to 90 days was calculated in a shift analysis. Ordinal logistic regression was used to identify factors associated with an increase in mRS points. Variables for regression models were selected using Akaike information criterion (AIC)-based backward selection. Odds ratios (OR) with 95% CI and *p*-values were calculated for selected variables. The effect of i.v. thrombolysis on the probability of reaching good clinical outcome (mRS ≤ 2) in M2 occlusions was furthermore evaluated in a logistic regression analysis stratified for administration of i.v. thrombolysis and successful recanalization, controlled for identified relevant covariates. Results including 95% CI were plotted with NIHSS at admission as independent variable.

## Results

**Figure 1:**
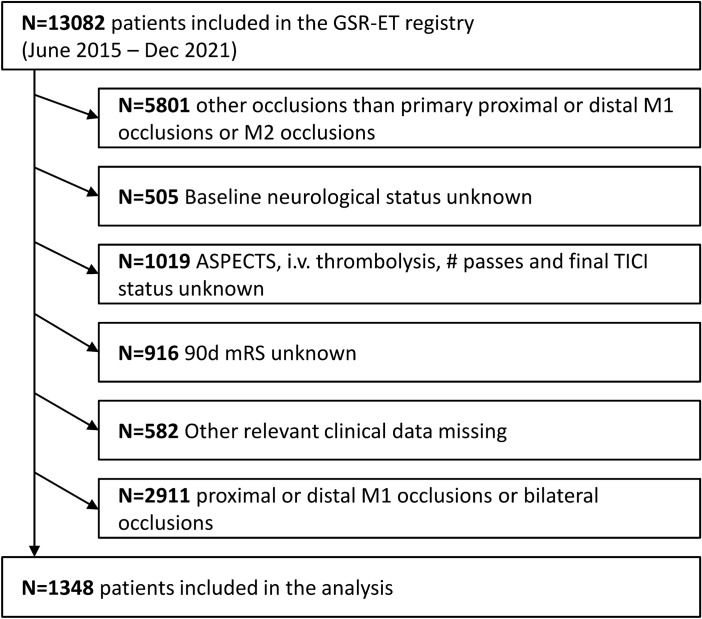
Patient inclusion flow chart. ASPECTS: Alberta Stroke Program Early CT Score, GSR-ET: German Stroke Registry-Endovascular Treatment, mRS: modified Rankin Scale, TICI: modified Thrombolysis in cerebral infarction.

1348 patients fulfilled the inclusion criteria (Fig. 1). 1129 (84%) patients received a successful recanalization, 407 (30%) had excellent functional outcome and 595 (44%) had good functional outcome, while poor outcome and death was recorded for 402 (30%) of patients. Results of the descriptive statistical analysis are shown in Table 1. Patients with excellent (90d mRS 0–1) and good outcome (90d mRS 0–2) were younger (71y and 72y vs. 79y and 80y, *p* < 0.001), were less often treated with anti-coagulation (35% and 37% vs. 48% and 49%, *p* < 0.001), had a lower pre-stroke mRS (*p* < 0.001), had a lower NIHSS at admission (7 vs. 12, *p* < 0.001), had less often preexisting hypertension (71% and 72% vs. 82% and83%, *p* < 0.001), had less diabetes (16% and 17% vs. 26% and 27%, *p* < 0.01), had less preexisting atrial fibrillation (33% and 37% vs. 50% and 51%, *p* < 0.001), had higher ASPECTS (10 and 9 vs. 9 and 9, *p* < 0.01), were more often treated with thrombolysis (61% and 57% vs. 42% and 41%, *p* < 0.001), had more often conscious sedation instead of general anesthesia (30% and 28% vs. 22% and 22%, *p* < 0.01)were more often recanalized successfully (92% and 93% vs. 80% and 77%, *p* < 0.001) with fewer passes (1.7 and 1.7 vs. 2.1 and 2.2, *p* < 0.001). Time from symptom onset/last seen well to groin puncture was lower in patients with excellent (223 min vs. 250 min, *p* < 0.01) and good functional outcome (231 min vs. 248 min, *p* < 0.05). mRS increase pre-stroke to 90d was lower with + 0 and + 1 versus + 3 and + 3, *p* < 0.001.

**Table 1:** Clinical data of study cohort by outcome.

	Excellent outcome (90d mRS 0–1)			Good outcome (90d mRS 0–2)			Poor outcome (90d mRS 5–6)			Total
	Yes	No		Yes	No		Yes	No		
	N = 407	N = 941	*p*-value	N = 595	N = 753	*p*-value	N = 402	N = 946	*p*-value	N = 1348
Age			< 0.001			< 0.001			< 0.001	
Median	71	79		72	80		82	75		77
Q1, Q3	60, 79	71, 85		61, 80	72, 85		75, 86	65, 82		68, 83
Sex (m)	228 (56%)	442 (47%)	0.002	319 (54%)	351 (47%)	0.011	177 (44%)	493 (52%)	0.007	670 (50%)
Anti thrombotic medication	141 (35%)	448 (48%)	< 0.001	218 (37%)	371 (49%)	< 0.001	204 (51%)	385 (41%)	< 0.001	589 (44%)
Pre-stroke mRS			< 0.001			< 0.001			< 0.001	
Median	0	0		0	1		1	0		0
Q1, Q3	0, 0	0, 2		0, 0	0, 2		0, 3	0, 1		0, 1
Baseline NIHSS			< 0.001			< 0.001			< 0.001	
Median	7	12		7	12		14	8		10
Q1, Q3	4, 11	7, 16		5, 11.5	8, 17		9, 18	5, 13		6, 15
Hypertension	287 (71%)	768 (82%)	< 0.001	427 (72%)	628 (83%)	< 0.001	341 (85%)	714 (75%)	< 0.001	1055 (78%)
Diabetes	66 (16%)	242 (26%)	< 0.001	102 (17%)	206 (27%)	< 0.001	116 (29%)	192 (20%)	< 0.001	308 (23%)
Dyslipidemia	195 (48%)	441 (47%)	0.724	272 (46%)	364 (48%)	0.338	183 (46%)	453 (48%)	0.426	636 (47%)
Atrial fibrillation	134 (33%)	466 (50%)	< 0.001	219 (37%)	381 (51%)	< 0.001	211 (52%)	389 (41%)	< 0.001	600 (45%)
Infarct side right	179 (44%)	386 (41%)	0.312	254 (43%)	311 (41%)	0.608	163 (41%)	402 (42%)	0.507	565 (42%)
ASPECTS			< 0.001			< 0.001			0.050	
Median	10	9		9	9		9	9		9
Q1, Q3	8, 10	8, 10		8, 10	8, 10		8, 10	8, 10		8, 10
i.v. thrombolysis	248 (61%)	398 (42%)	< 0.001	340 (57%)	306 (41%)	< 0.001	151 (38%)	495 (52%)	< 0.001	646 (48%)
Treatment in admission center	295 (74%)	615 (67%)	0.010	406 (70%)	504 (69%)	0.484	278 (71%)	632 (69%)	0.479	910 (69%)
Anesthesia			0.003			0.008			0.013	
Local, change to general	12 (3%)	43 (5%)		17 (3%)	38 (5%)		20 (5%)	35 (4%)		55 (4%)
Conscious sedation	119 (30%)	202 (22%)		161 (28%)	160 (22%)		76 (19%)	245 (27%)		321 (24%)
General anesthesia	260 (66%)	680 (74%)		400 (69%)	540 (73%)		299 (76%)	641 (70%)		940 (71%)
Number of passes			< 0.001			< 0.001			0.001	
Mean (SD)	1.7 (1.2)	2.1 (1.6)		1.7 (1.2)	2.2 (1.7)		2.2 (1.6)	1.9 (1.4)		2.0 (1.5)
Q1, Q3	1, 2	1, 3		1, 2	1, 3		1, 3	1, 2		1, 3
Final TICI ≥ 2b	375 (92%)	754 (80%)	< 0.001	551 (93%)	578 (77%)	< 0.001	286 (71%)	843 (89%)	< 0.001	1129 (84%)
Final TICI = 3	233 (57%)	435 (46%)	< 0.001	331 (56%)	337 (45%)	< 0.001	156 (39%)	512 (54%)	< 0.001	668 (50%)
Symptom onset to groin puncture (min)			0.002			0.034			0.092	
Median (SD)	223	250		231	248		255	235		240
Q1, Q3	145, 422	155, 470		155, 430	165, 474		170, 519	155, 432		160, 451
Dissection or perforation	8 (2%)	38 (4%)	0.054	14 (2%)	32 (4%)	0.057	23 (6%)	23 (2%)	0.002	46 (3%)
Clot migration or embolisation	12 (3%)	40 (4%)	0.254	20 (3%)	32 (4%)	0.400	16 (4%)	36 (4%)	0.879	52 (4%)
Intracranial hemorrhage	13 (3%)	51 (5%)	0.078	15 (3%)	49 (7%)	< 0.001	30 (7%)	34 (4%)	0.002	64 (5%)
sICH 24 h	1 (0.2%)	49 (5.2%)	< 0.001	3 (0.5%)	47 (6.2%)	< 0.001	40 (10%)	10 (1.1%)	< 0.001	50 (3.7%)
Vasospasm	23 (6%)	53 (6%)	0.989	33 (6%)	43 (6%)	0.897	23 (6%)	53 (6%)	0.931	76 (6%)
90d mRS			< 0.001			< 0.001			< 0.001	
Median	1	4		1	5		6	2		3
Q1, Q3	0, 1	3, 6		0, 2	3, 6		6, 6	1, 3		1, 5
Pre-stroke to 90d mRS change			< 0.001			< 0.001			< 0.001	
Median	0	3		1	3		5	1		2
Q1, Q3	0, 1	2, 4		0, 1	3, 5		3, 6	0, 2		1, 4

Patients with poor outcome or death (90d mRS 5–6) were older (82y vs. 75y, *p* < 0.001), had a higher pre-stroke mRS (1 vs. 0, *p* < 0.001), had a higher NIHSS at admission (14 vs. 8, *p* < 0.001), were more often treated with anti-thrombotic medication (51% vs. 41%, *p* < 0.001) had more often preexisting hypertension (85% vs. 75%, *p* < 0.001) and diabetes (29% vs. 20%, *p* < 0.001), had more often atrial fibrillation (52% vs. 41%, *p* < 0.001), received less often i.v. thrombolysis (38% vs. 52%, *p* < 0.001), had more passes (2.2 vs. 1.9, *p* < 0.001), were less often recanalized successfully (71% vs. 89%, *p* < 0.001), had more dissections and perforations during treatment (6% vs. 2%, *p* < 0.01) and had higher rates of ICH during treatment (7% vs. 4%, *p* < 0.03) as well as higher rates of sICH at 24h (10% vs. 1%, *p* < 0.001). mRS-increase pre-stroke to 90d was higher with + 5 versus + 1, *p* < 0.001.

In patients with excellent outcome (90d mRS 0–1) the share of females was significantly lower (44% vs. 53%, *p* < 0.01), while in patients with poor outcome (90d mRS 5–6) the share of females was significantly higher (56% vs. 48%, *p* < 0.01). Furthermore, in patients with excellent outcome, the share of patients treated in the admission center was higher (74% vs. 67%, *p* < 0.01).

**Figure 2:**
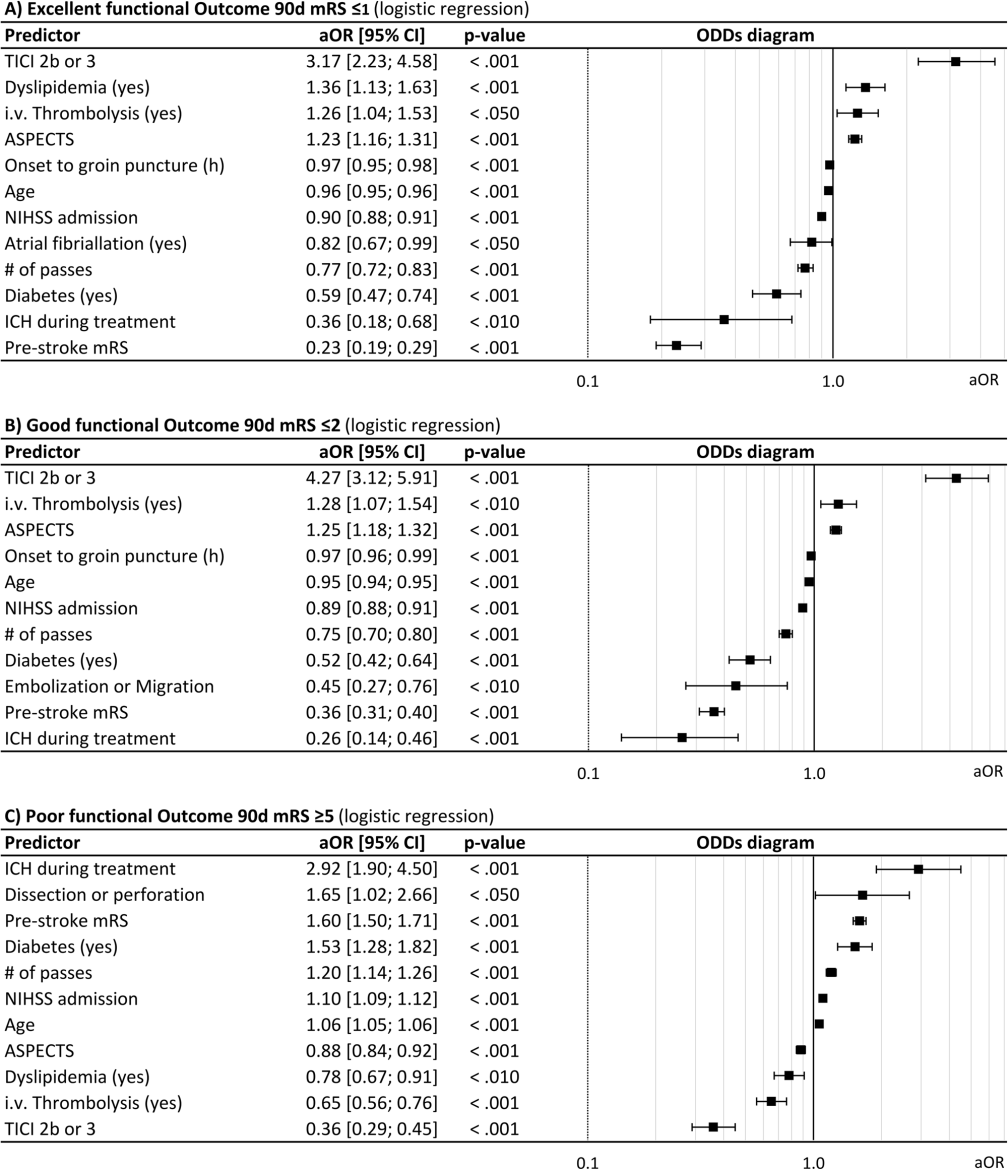
Predictors of excellent, good and poor functional outcome after thrombectomy for M2 occlusions, multivariable logistic regression analysis. AE: Adverse events, ASPECTS: Alberta Stroke Program Early CT Score, CI: Confidence interval, mRS: modified Rankin Scale, NIHSS: National Institute of Health Stroke Scale, OR: Odds ratio, TICI: modified Thrombolysis in cerebral infarction.

In multivariable logistic regression analysis (Fig. 2), probability of good outcome (mRS ≤ 2) was reduced by higher age (odds ratio [OR] 0.95 [95% confidence interval 0.94–0.95], *p* < 0.001), higher pre-stroke mRS (OR 0.36 [0.31–0.4], *p* < 0.001), higher baseline NIHSS (OR 0.89 [0.88–0.91], *p* < 0.001), comorbidity diabetes (OR 0.52 [0.42–0.64], *p* < 0.001), higher number of passages (OR 0.75 [0.70–0.80], *p* < 0.001), longer times from symptom onset/last seen well to groin puncture (OR 0.97 [0.96–0.99], *p* < 0.001), embolization and clot migration (OR 0.45 [0.27–0.76], *p* < 0.01) and intracranial hemorrhage (OR 0.26 [0.14–0.46], *p* < 0.001), while higher ASPECTS (OR 1.25 [1.18–1.32], *p* < 0.01), i.v. thrombolysis (OR 1.28 [1.07–1.54], *p* < 0.01) and successful recanalization (OR 4.27 [3.12–5.91], *p* < 0.001) increased the probability of good outcome. Correspondingly, probability of excellent outcome (mRS ≤ 1) was reduced with higher age (OR 0.96 [0.95–0.96], *p* < 0.001), ), higher pre-stroke mRS (OR 0.23 [0.19–0.29], *p* < 0.001), higher baseline NIHSS (OR 0.90 [0.88–0.91], *p* < 0.001), diabetes (OR 0.59 [0.47–0.74], *p* < 0.001), intracranial hemorrhage (OR 0.36 [0.18–0.68], *p* < 0.01), longer times from symptom onset/last seen well to groin puncture (OR 0.97 [0.95–0.98], *p* < 0.001) and higher number of passages (OR 0.77 [0.72–0.83], *p* < 0.001), while higher ASPECTS (OR 1.23 [1.16–1.31], *p* < 0.01), i.v. thrombolysis (OR 1.26 [1.04–1.53], *p* < 0.05) and successful recanalization (OR 3.17 [2.23–4.58], *p* < 0.001). In addition, excellent functional outcome was associated with dyslipidemia (OR 1.36 [1.13–1.63], *p* < 0.001), while atrial fibrillation (OR 1.82 [0.67–0.99], *p* < 0.05) reduces probability of excellent outcome.

Predictors of poor outcome and death after 90 days were higher age (OR 1.06 [1.05–1.06], *p* < 0.001), higher baseline mRS (OR 1.60 [1.50–1.71], *p* < 0.001), higher NIHSS at admission (OR 1.10 [1.09–1.12], *p* < 0.001), diabetes (OR 1.53 [1.28–1.82], *p* < 0.001), higher number of passages (OR 1.20 [1.14–1.26], *p* < 0.001), dissections and perforations (OR 1.65 [1.02–2.66], *p* < 0.05) and intracranial hemorrhage (OR 2.92 [1.90–4.50], *p* < 0.001). i.v. thrombolysis (OR 0.65 [0.56–0.76], *p* < 0.01), dyslipidemia (OR 0.78 [0.67–0.91], *p* < 0.01), higher ASPECTS (OR 0.88 [0.84–0.92], *p* < 0.001) and successful recanalization (OR 0.36 [0.29–0.45], *p* < 0.001) reduced risk of poor outcome.

**Figure 3:**
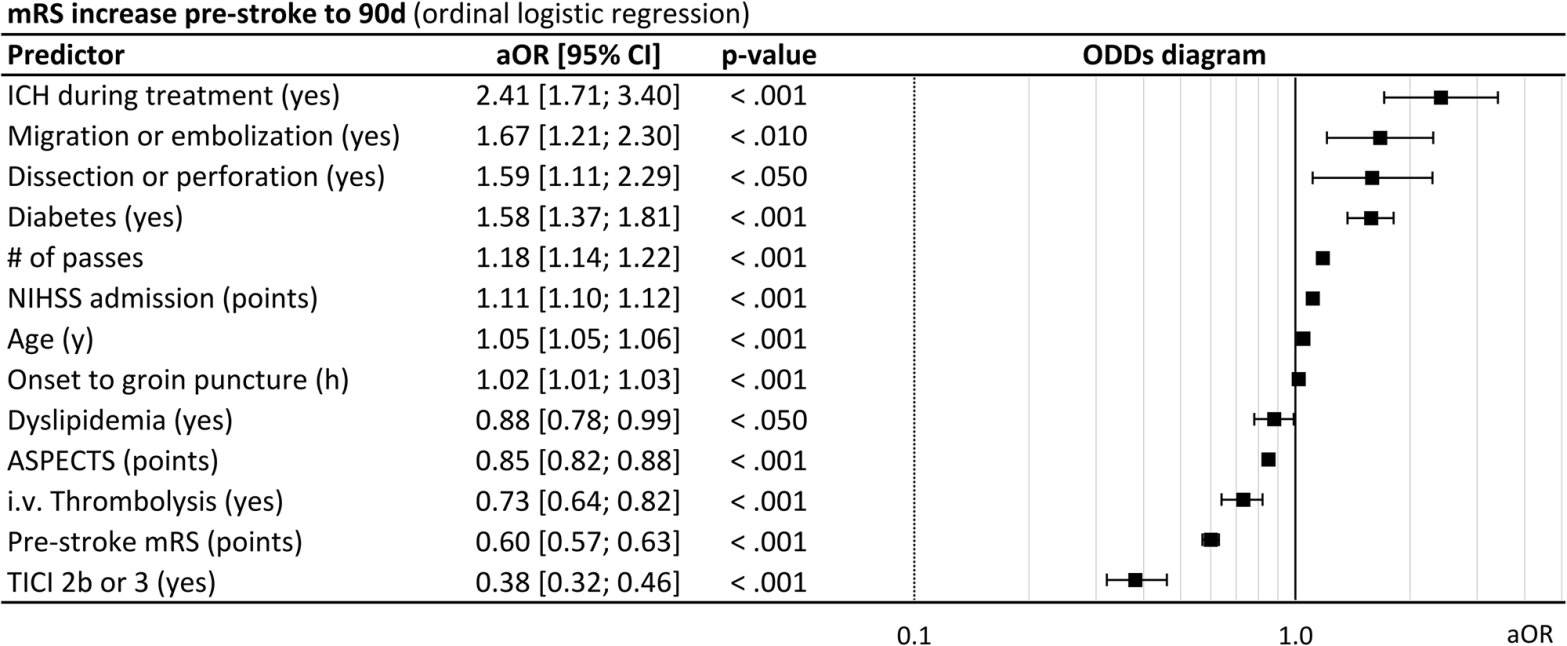
Predictors of mRS increase pre-stroke to 90d after thrombectomy for M2 occlusions, multivariable ordinal logistic regression analysis. AE: Adverse events, ASPECTS: Alberta Stroke Program Early CT Score, CI: Confidence interval, mRS: modified Rankin Scale, NIHSS: National Institute of Health Stroke Scale, OR: Odds ratio, TICI: modified Thrombolysis in cerebral infarction.

Results from ordinal logistic on mRS increase pre-stroke to 90d suggest that higher age (OR 1.05 [1.05–1.06], *p* < 0.001), higher baseline NIHSS (OR 1.11 [1.10–1.12], *p* < 0.001), comorbidity diabetes (OR 1.53 [1.28–1.82], *p* < 0.001), higher number of passes (OR 1.18 [0.14–0.22], *p* < 0.001) and longer time from symptom onset/last seen well to groin puncture (OR 1.02 [1.01–1.03], *p* < 0.001) increase the risk of a mRS gain (per point). Furthermore, treatment related complications dissections and perforation (OR 1.59 [1.11–2.29], *p* < 0.05), clot migration and embolization (OR 1.67 [1.21–2.30], *p* < 0.01) and intracranial hemorrhage (OR 2.41 [1.71–3.40], *p* < 0.001) were associated with mRS gain pre-stroke to 90d. In contrast, lower baseline mRS (OR 0.60 [0.57–0.63], *p* < 0.001), i.v. thrombolysis (OR 0.73 [0.64–0.82], *p* < 0.001), dyslipidemia (OR 0.88 [0.78–0.99], *p* < 0.001) and successful recanalization (OR 0.38 [0.32–0.46], *p* < 0.001) were associated with lower risk of further neurological deterioration (Fig. 3).

**Figure 4:**
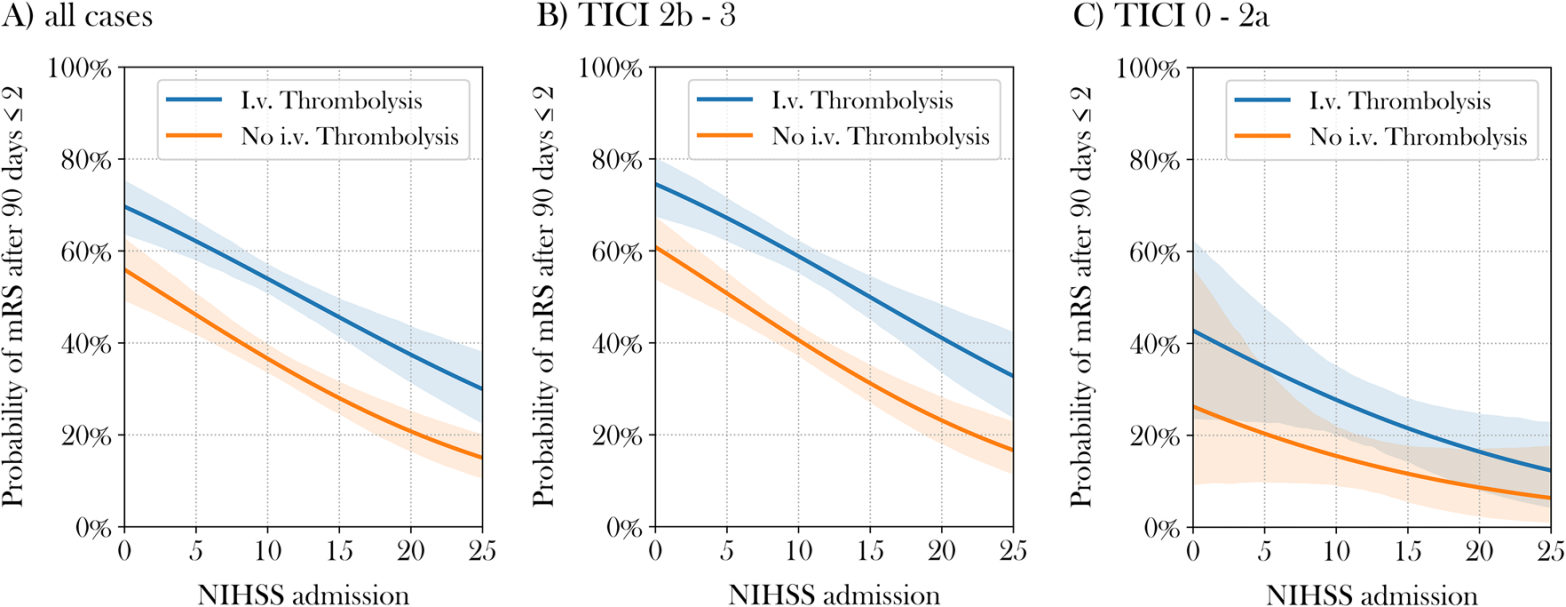
Probability of good outcome depending on recanalization status and i.v. thrombolysis in multivariable logistic regression analysis. All analysis were controlled for Age, pre-mRS, Diabetes, ASPECTS and number of passes. NIHSS: National Institute of Health Stroke Scale, mRS: modified Rankin Scale, TICI: modified Thrombolysis in cerebral infarction.

Logistic regression analysis stratified for i.v. thrombolysis and recanalization success suggests that administration of i.v. thrombolysis increases probability of good outcome for all levels of initial neurological impairment. (Fig. 4A). Differentiation by recanalization success confirms these findings for patients with successful MT. At NIHSS at admission of 10, an increase of probability of good outcome from c. 40% to c. 60% was observed, equaling a 33% reduction of relative risk (Fig. 4B). In cases with TICI 0 to TICI 2a, i.v. thrombolysis might improve prognosis especially for patients with mild symptoms at admission, however, differences are not significant (Fig. 4C).

## Discussion

Our registry study based on large scale clinical experience from prospectively enrolled patients showed that an older age, a higher baseline mRS, higher NIHSS at admission, preexisting diabetes and higher number of passes were strongly associated with lower chances of good clinical outcome and even higher risk of poor outcome and death at 90 days after MT for M2 occlusions. In comparison, a successful recanalization (TICI ≥ 2b) and i.v. thrombolysis were associated with higher chances of good clinical outcome at 3 months and a reduced risk for poor outcome. Ordinal logistic regression on mRS shift pre-stroke to 90 days suggests that treatment related complications like dissections, perforations, clot migration and embolization and intracranial hemorrhage are associated with higher mRS increase pre-stroke to 90d.

Descriptive statistics show that in patients with excellent outcome the share of females was significantly lower while in patients with poor outcome the share of females was significantly higher. However, this observation was not reflected in results of the multivariable regression analysis, suggesting that in our cohort female strokes might be associated with other risk factors, such as higher age.

Furthermore, in patients with excellent outcome, the share of patients treated in the admission center was higher.

Compared to results of recently published meta-analysis of randomized controlled trials including n = 195 patients receiving MT for M2 occlussions^9^, our clinical cohort was of higher age (77y vs. 71y) with similar rates of hypertension, dyslipidemia, atrial fibrillation and diabetes. Median NIHSS at admission in our cohort was 10 compared to 13 in the meta-analysis. However, our results suggest a lower rate of excellent outcome with 30% versus 52% and a lower rate of good outcome with 44% versus 68% in the RCT cohort. However, a direct comparison of functional outcomes is not possible as the authors did not provide information about recanalization success in the RCT cohort.

Predictors of good clinical outcome have been evaluated in MT for acute ischemic stroke with LVO^15–19^. Identified predictors for good clinical outcome in M2 occlusions are comparable to those suggested for LVO^15,20^. As previously reported by Wollenweber et al., predictors of good clinical outcome included younger age, smaller infarct size, lower stroke severity, alteplase use and reperfusion success. Correspondingly, in our cohort, younger age, higher ASPECTS, lower NIHSS at admission and successful recanalization were strongly associated with higher chances of good clinical outcome at 90 days after a MT for M2 occlusions. In comparison, Hulscher et al.^10^ did not identify admission NIHSS as a predictor of poor functional outcome in medium vessel occlusion patients treated with MT, while another study by Mokin et al.^11^ did find an association between baseline NIHSS and functional outcome. This association might therefore be more dependent on specific cohort characteristics, such as predefined inclusion criteria and center-specific treatment decisions^21^.

Recanalization rates of 84% in our study were slightly higher in comparison with previous reports of successful recanalization (final recanalization TICI score ≥ 2b) of 78% in a meta-analysis of M2 occlusions^22^. As it is known from LVO studies, our data also suggest that a higher number of recanalization attempts reduces the chance of good clinical outcome and even increases the chance for poor outcome after 90 days in MT for M2 occlusions^23^. The effect of the number of passes regarding safety and efficiency to recanalize medium vessel occlusions needs to be investigated in multicentric and larger cohorts.

It has been shown that one third of medium vessel occlusion stroke patients have a baseline NIHSS < 6^24^. In our study only c. 20% of the patients presented with an NIHSS < 6. The subgroup of patients with mild symptoms was therefore smaller than expected. This can be explained by the fact that patients from the underlying registry data are not selected using pre-defined inclusion criteria and the decision to treat patients with M2 occlusion was made by the treating neurointerventionalist or neurologist. While cases with M2 occlusion and low clinical severity could more likely be managed only medically (and would therefore not be included in the registry), primary M2 occlusions with severe baseline NIHSS might be associated with higher willingness of neurointerventionalists for endovascular thrombectomy^21,25^.

Good clinical outcome at 90 days was achieved in 44% of the patients included in our study, which is below the range of rates reported in the literature for M2 occlusions between 56%^11^ and 62%^22^ but higher than rates reported for M1 occlusions.

Our results suggest that comorbidity diabetes is associated with lower chance of good outcome and increased risk of neurological deterioration. As diabetes is associated with microvascular dysfunction, higher rates of microangiopathy-related complications during treatment and post-interventional adverse events might be an explanation for our observations. It has been shown that higher blood glucose levels are associated with worse clinical outcome^26^, but the pathophysiological pathways are not fully understood yet. Recent studies suggest that higher blood glucose levels trigger increased edema formation and might thereby lead to worse functional outcome^27,28^. However, other pathways have been discussed as well: Higher serum glucose levels could alter vessel intima tissue and thrombus characteristics and lead to increased thrombogenicity. Also, higher serum glucose levels could negatively influence cellular metabolism and ischemic tolerance.

Furthermore, dyslipidemia was associated with higher probability of excellent outcome and lower risk for poor outcome and mRS increase pre-stroke to 90d. Although the role of dyslipidemia on recanalization success is not investigated in detail yet, it can be speculated that statins with their known positive effects on inflammatory processes in vessel walls might increase chances of successful recanalization and better prognosis.

It has previously been emphasized that the NIHSS and mRS scales are limited in their granularity and heavily focused on motor function and thus, unable to capture domain-specific impairment that often play a dominant role in medium vessel occlusion stroke-related disability^24^. Furthermore, based on a smaller area that is affected by ischemia, clinical outcomes can be expected to be better than in LVO stroke patients. Given the overall better prognosis, it has been proposed to consider more restrictive outcome measure such as excellent outcome or mRS shift analysis^24^. We therefore also evaluated predictors significantly associated with neurological deterioration based on point-wise shift in mRS to increase the granularity of the outcome measure and capture associations that might otherwise not be captured. Results suggest that treatment related complications are not fully captured by binarized outcome metrics but are significantly associated with mRS increase pre-stroke to 90d.

Results furthermore suggest that administration of i.v. thrombolysis in patients with M2 occlusions even in combination with MT might improve outcome, apart from the already assumed higher effectiveness of pharmacologic fibrinolysis for the smaller clot burdens of medium vessel occlusions compared to larger clot burdens of LVO^29–31^.

Our study has limitations. All clinical parameters including mRS, vessel occlusion status and location were site reported parameters that might suffer from site related bias due to limited interrater reliability. The study cohort was selected based on center assessments of the exact occlusion location. It is known that the exact anatomical M2 definition is a matter of debate. For MCA occlusions, the GSR-ET registry differentiates proximal M1, distal M1 and M2 occlusions. The study aims to identify factors leading to good or poor outcome in patients undergoing MT with medium and distal occlusions of the MCA in comparison to M1 occlusions, the study does not aim to proof safety and efficiency of a specific therapy. Due to numerous anatomical variants of the MCA, cohort definitions with exactly the same branching and vessel diameter parameters might not be possible. In addition, such cohort selected by narrow criteria would only reflect specific study settings but would not reflect clinical practice where treating physicians typically differentiate proximal, medium and distal vessel occlusions based on less narrow/specific criteria. Therefore, evaluations of outcomes of medium and distal vessel occlusions might be better generalizable to clinical practice if the in- and exclusion criteria of the study cohort match with the understanding and conduct in clinical practice. It therefore can be assumed that prospective registry data of real clinical cases ensures high consistency and generalizability of reported results to clinical practice. The registry does not include perfusion imaging results and collateral status, which are both predictors of outcome in ischemic stroke. However, perfusion imaging is not included in the standard of care in many centers. In addition, it has been shown that the predefined thresholds for penumbra and core segmentations might not reflect final infarct cores. However, the analysis includes ASPECTS scoring, which has been shown to be a valid predictor of outcome and a surrogate for the extend of the final infarct core.

Furthermore, only cases with availability of all required data points were included in the analysis. Exclusion of patients with missing data points (including lost to follow-up) might introduce bias to the reported results and might reduce generalizability of findings. Results from RCTs are needed to confirm our observations.

Three RCTs currently upcoming and enrolling (DISTAL, ESCAPE-MEVO and FRONTIER-AP) will further enhance the understanding of safety and efficiency of MT in medium and distal vessel occlusions.

## Conclusion

Corresponding to results reported for LVO, younger age, low pre-stroke mRS, low severity of acute clinical disability, i.v. thrombolysis and successful recanalization were associated with good outcome while preexisting disability, diabetes and higher number of retrieval attempts decreased the probability of reaching a good functional status after MT for M2 occlusions. Treatment related complications were associated with higher risk of mRS shift towards increased neurological impairments versus pre-stroke.

## Data Availability

The data that support the findings of this study are available from the GSR-ET registry, but restrictions apply to the availability of these data, and so the data is not publicly available. Data are available from the corresponding author upon reasonable request and with permission of the GST-ER registry.
